# Factors governing consumers buying behavior concerning nutraceutical product

**DOI:** 10.1002/fsn3.3518

**Published:** 2023-06-27

**Authors:** Harsh B. Jadhav, Shyam Sablani, Parag Gogate, Uday Annapure, Federico Casanova, Gulzar Ahmad Nayik, Kamal Alaskar, Nazmul Sarwar, Irfan Ahmad Raina, Seema Ramniwas, Amin Mousavi Khaneghah

**Affiliations:** ^1^ Department of Food Engineering and Technology Institute of Chemical Technology Mumbai India; ^2^ Biological Systems Engineering Department Washington State University Pullman Washington USA; ^3^ Department of Chemical Engineering Institute of Chemical Technology Mumbai India; ^4^ Research Group for Food Production Engineering, National Food Institute Technical University of Denmark Kongens Lyngby Denmark; ^5^ Department of Food Science and Technology Government Degree College Shopian Jammu & Kashmir India; ^6^ Department of Computer Application Bharathi Vidyapeeth Institute of Management Kolhapur, Pune Maharashtra India; ^7^ Department of Food Processing and Engineering Chattogram Veterinary and Animal Sciences University Chattogram Bangladesh; ^8^ Department of Food Science and Technology University of Kashmir Srinagar Jammu & Kashmir India; ^9^ University Centre for Research and Development Chandigarh University Mohali Punjab India; ^10^ Department of Fruit and Vegetable Product Technology Prof. Wacław Dąbrowski Institute of Agricultural and Food Biotechnology – State Research Institute Warsaw Poland; ^11^ Department of Technology of Chemistry Azerbaijan State Oil and Industry University Baku Azerbaijan

**Keywords:** buying behavior, consumer behavior, dietary supplement, functional foods, market size, nutraceutical

## Abstract

In recent years, consumers are increasingly attracted to nutraceuticals, an important part of food considered propitious for human health. Therefore, consumers are willingly switching to nutraceuticals and are ready to pay the premium price. This review aims to identify various factors that govern consumer purchasing of nutraceutical products. The outcomes presented in the review provide a closer understanding of consumer attitudes toward buying behavior and their impact on the growth of the global nutraceutical market. The nutraceutical market has been identified depending on the type of nutraceuticals, forms, and regions governing the nutraceutical market. Factors such as health consciousness, knowledge about a product, product availability, price, marketing strategies, and social factors influence consumers' actual buying behavior toward nutraceutical products. A mini survey in Mumbai city of India was conducted to add practical data to the review, and factors affecting consumers' willingness to buy nutraceutical products were identified. It was observed that the decision‐making toward buying nutraceutical products was affected by gender, age, education level, and acculturation. It was also identified that the legislation governing nutraceuticals needs to be harmonized throughout many parts of the world, which restricts the growth of this sector to some extent. The findings elucidate that nutraceutical industries should overcome the regulatory barriers and focus on developing innovative products, which will keep current consumers intact and help increase the consumer base and thus expand the nutraceutical market globally.

## Graphical abstract

The findings in this article elucidate that nutraceutical industries should overcome the regulatory barriers and focus on the development of innovative products, which will not only keep current consumers intact but will also help increase the consumer base and thus expand the nutraceutical market globally. In summary, nutraceutical industries should adopt simple and informative advertisement patterns and also they should work on innovative products which could be easy to carry and easy to eat.

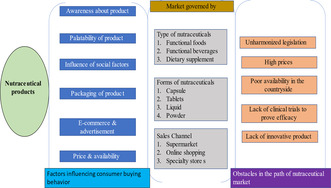

## 1 INTRODUCTION

A sedentary lifestyle and lack of a nutritious diet have resulted in an epidemic of chronic diseases such as obesity, anxiety, depression, heart diseases, diabetes, high blood pressure, cancer, and weak immune system (Park et al., 2020; Ssewanyana et al., 2018). To overcome this, consumers are demanding food that will not only satisfy hunger but will also benefit consumers by preventing health‐related diseases and will keep them physically and mentally well (Azeredo et al., 2021; Dominguez‐Viera et al., 2022). Consumer expectations have burdened food industries with delivering food products with additional health benefits (Nwosu & Ubaoji, 2020). The demand has gained momentum globally in the COVID‐19 pandemic (Lordan, 2021). The names like ‘nutraceuticals,’ ‘dietary supplement,’ ‘probiotic,’ ‘functional foods,’ and ‘organic food’ have attracted the attention of consumers lately and are becoming popular among consumers of all age groups and are commonly contributing to health‐related food (HRF) segment of the food industry. Though different names know them, they aim to improve human health while satisfying hunger. The health‐related food industry is continuously expanding and experiencing dynamic growth every day. The industries are developing new and innovative products to meet increasing demand (Gutkowska & Czarnecki, 2020; Herath et al., 2008; Jadhav & Annapure, 2021; Siegrist et al., 2015).

Nutraceuticals came into the limelight in the late eighties and attracted the interest of food scientists and technologists after that. Nutraceuticals typically include functional foods, nutritional supplements, and probiotics (Nwosu & Ubaoji, 2020). The nutraceutical sector is growing, but this growth is arrested by factors such as consumer attitude, lack of clinical evidence, and strict regulations, which are responsible for slow expansion in this sector. Consumer buying behavior plays a vital role in the growth of health‐food‐related sectors, including the nutraceutical sector. Due to a lack of awareness, consumers need clarification about whether to consider nutraceuticals as food or medicine and are skeptical about overstated health claims. The regulations governing nutraceuticals differ in each country, affecting the market's growth. For instance, in Europe, nutraceutical products need certification from European Food Safety Authority, which checks product claims by doing various tests, and similarly, in Canada, the same procedure is used. In both these cases, little claims are approved while others not provided scientifically are ruled out, which affects the market's growth. The USA is the largest market for nutraceuticals, followed by Japan. In the US, the products are subjected to various government norms if any nutraceutical product is claimed to treat any disease (Ahmad et al., 2011; Nwosu & Ubaoji, 2020). They must pass various tests to get into the market. In developing countries like India, where the nutraceutical industry has a huge future, strict rules for product launches exist as laid out by the Food Safety and Standard Authority of India (FSSAI). The absence of synchronized regulations can act as a resistance to the growth of the nutraceutical sector across the globe (Stirling & Kruh, 2015).

Consumer behavior has changed due to the COVID pandemic, as holistic health has become a focal point of the global population (Bhagra et al., 2020). People have started realizing the importance of nutraceuticals in keeping the body fit and enhancing the immune system to fight against infections like COVID, changing the consumer mind from curative to preventive well‐being (Ratha et al., 2021; Shamsudin et al., 2022). Not only COVID but also lifestyle change has been a reason for the emergence of lifestyle‐related diseases. To overcome this, consumers have started changing their dietary habits. The consumer has also realized that improper diet will directly affect their health and may result in hospitalization, where the consumer has to bear the huge hospitalization cost, which also puts an extra burden on the healthcare system. This has driven consumers toward nutraceuticals as precautionary measures rather than spending huge amounts on treatment. The metamorphosis in consumer behavior due to lifestyle changes and increased consciousness transfigures the nutraceutical market in developed and developing economies. The COVID pandemic has further boosted the demand for nutraceuticals leading to a blossoming market for nutraceuticals.

Due to the increase in consumer demand, even during the COVID pandemic, the global nutraceutical market has grown significantly, with the market size increasing from USD 320 billion in 2020 to USD 352.92 billion in 2021 (Lordan, 2021). It is expected to increase to USD 658.11 billion by the end of 2028, with a compound annual growth rate of more than 9%. Asia Pacific holds a remarkable contribution to the nutraceutical market. It is expected to emerge as a global leader in the nutraceutical sector due to consumer awareness and the rising population in countries like China (Tripathi et al., 2020). North America is expected to be the second largest nutraceutical market due to growth in regional markets where consumers have a high demand for nutraceuticals like health drinks, dietary supplements, and fortified/functional drinks. Even in Europe, the sector has experienced a boom in dietary supplements. Nutraceutical players here are focusing on developing new and innovative nutraceuticals to capture the market and meet the needs of consumers. For example, a Brazilian company developed a new dietary supplement, ‘MENA Q7 VITAIN K2’ in 2019, targeting old age population. In some parts of the world, like South America, the nutraceutical sector is in its early stage and contributes only 12% to the global nutraceutical market. It is expected that this will increase in the years to come depending on the consumer acceptance of nutraceutical products and how big industrial players deliver it to their targeted group. In India, international nutraceutical industries such as Unilever, Glaxo Smith Kline, Amway, Danone, Nestle, and Kellogg's are coming up with nutraceutical products to cater to the need of the Indian population. Apart from this, many Indian players like Dabur, Himalaya, and Patanjali have also come forward with nutraceutical products and trying to reach every segment of the country to grab their share. India has eloquently younger consumers who are vigorously focusing on active lifestyles and aiming to avoid lifestyle‐related diseases like obesity and diabetes. India will emerge as a hub for the nutraceutical sector, which will experience accelerated growth in the Indian market. This review aims to highlight consumer behavior while choosing nutraceutical products and critically review all the accessible evidence to recognize consumers' response toward nutraceutical products and how this behavior has been a driving force to boost the nutraceutical sector. The forms of nutraceuticals ruling the global nutraceutical market and the regulations governing it have been elucidated. A mini survey in Mumbai city of India was conducted to identify the need of the local population and their hopes for the nutraceutical sector have also been presented. Mumbai is one of the fastest‐growing metro cities in India, with a total population of 17,159,000 and with 89.73% literacy rate in 2023. The total literate population in Mumbai is 10,084,507, of which 5,633,709 are males while 4,450,798 are females (Population Census, 2023). In the third decade of the 21st Century, health has become consumers' priority from societal and personal perspectives. Hence, comprehending these elements will be useful for the nutraceutical sector to concoct a marketing strategy for expanding the nutraceutical sector globally.

## 2 NUTRACEUTICALS

**FIGURE 1:**
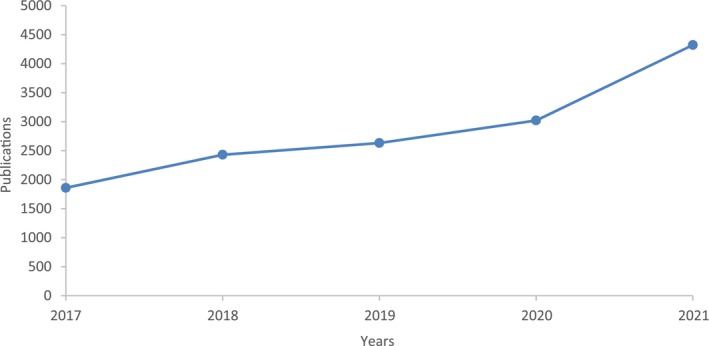
Increase in number of research publications over the last 5 years (Scopus was used to get the number of publications based on queries like functional foods, nutraceuticals, functional beverages, and dietary supplements).

Nutraceuticals is an emerging area between ‘Nutrition and Pharmaceuticals that has attracted consumers’ attention owing to its health‐friendly traits useful in preventing diseases. Both nutrition and pharmaceuticals have played a vital role in human wellness through the ages. The combined term nutraceutical came into the limelight only in the last 25 years and has helped prevent chronic diseases and improve human health (Garima & Manoj, 2016; Jha et al., 2021). A profusion of corroborations is available to support the benefits nutraceuticals deliver to keep human health well and sound. A simple definition of a nutraceutical is “a product which has the capability of preventing and treating disease and is also beneficial for human health” (Pandey et al., 2014). The term ‘nutraceutical’ is generally used for an extensive range of products like dietary supplements, functional foods, functional beverages, extracted bioactive, herbal products, and processed food containing nutritional components (Banwo et al., 2021; Sabra et al., 2021; Venhuis et al., 2016; Yilmaz‐akyuz et al., 2019). Due to their potential health benefits, nutraceuticals have attracted the attention of food scientists. They are among the most researched topics, evidenced by online scientific publications and increasing yearly (Figure 1). The growing attentiveness to nutraceuticals can also be seen from the growing market value, which is expected to cross USD 658.11 billion by the end of 2028. However, the nutraceutical sector still needs to work on getting into the mainstream of the food market. There are many obstacles in the growth of the nutraceutical sector, which has hampered the development and viability of this sector over a long period (Finley, 2016). The strong reason contributing to the obstacles is the complex and perplexing regulations on nutraceuticals, which are also different in different countries, preventing easy export of nutraceutical products among different nations. The regulations have put restrictions not only on developing new nutraceuticals but also on marketing claims, especially in terms of health benefits. Additionally, there is a state of incredulity among consumers regarding nutraceutical products. Because the data in the literature are obtained based on animal studies or in vitro trials of nutraceuticals, there is always a doubt that the observed effects or conclusions may not apply to humans. There is a lack of solid evidence which could strongly recommend using nutraceuticals for humans. A published study (Ioannidis, 2012) highlighted 525 animal studies, and out of so many studies, only one was true and efficacious. Hence, the nutraceutical industries should focus much on clinical trials to prove the claimed health benefits so that consumers in confused situations can clear their doubts, which will not only clear the path for growth but also add to the development and growth of the nutraceutical sector.

## 3 CONSUMERS BUYING BEHAVIOR – NUTRACEUTICAL PRODUCTS

The important factors governing consumers' behavior toward buying nutraceutical products are summarized based on the literature analysis presented in Table 1.

**TABLE 1:** Survey carried out in various countries to identify consumer's attitude toward nutraceutical products.

Objective of survey	Targeted consumers	Conclusion	References
To check consumers’ point of view toward nutraceuticals	Consumers in age group of 15–74 years from Finland	Consumers' attitude toward the use of nutraceuticals like functional foods was governed by perceived rewards and trust in nutraceutical products	Urala and Lähteenmäki (2004)
Consumer's acceptance of nutraceuticals	215 consumers in the age group of 15–50 years from Belgium	Consumers' attitude toward acceptance of nutraceuticals is governed by sociodemographic factors, Women and old age people showed higher acceptance of nutraceuticals	Verbeke (2005)
To check the effect of nutraceutical knowledge on buying behavior	Consumers in age group of 18–81 years (104 consumers) from Uruguay	Consumers with proper knowledge of nutraceuticals showed higher willingness to buy products, whereas consumers with inadequate knowledge about product showed less interest	Ares et al. (2008)
To check the effect of different health claims on purchasing behavior of consumers	Consumers above 18 years of age were selected from Australia (149 consumers)	Consumers are attracted toward nutraceutical products with health claims, especially that related to prevention of lifestyle‐related diseases	Williams et al. (2008)
To examine consumers' attitude toward health claim on nutraceutical products	178 and 207 students were respondents in two different surveys carried out in United States of America	Consumers were highly receptive to health claims made by the manufacturers. Survey revealed that consumers opted for nutraceutical products with health benefits more than 50% of the time	Naylor et al. (2009)
To investigate keenness of consumers toward nutraceutical products	An online survey was conducted in Germany and China. In Germany, a total of 502 participants joined, whereas in China 443 consumers participated	Consumers in China showed higher interest in buying nutraceutical products as compared with consumers in Germany. However, neophobia was higher in Chinese as compared with German consumers. The traditions also played an important role in buying of nutraceuticals	Siegrist et al. (2015)
To investigate the effect of social factors on willingness to purchase nutraceutical products	Survey was conducted among 695 consumers from Istanbul, Turkey	Consumers who are aware of the health benefits of nutraceuticals, who are accessible to media, and who are educated are more likely to buy nutraceutical products in Istanbul	Bekoglu et al. (2016)
To evaluate the role of sociodemographic factors in deciding the purchase of nutraceutical product	The survey was done among 137 female and 63 male consumers in the age group of 8–60 years from Holland	Consumers with age more than 50 years and women consumers were found to show much interest in buying nutraceutical products having health benefits	Kraus et al. (2017)
To investigate the attitude of consumers toward nutraceutical products and purchasing behavior	Survey was done in Spain among 333 consumers.	Consumer's attitude is directly related to their interest in buying nutraceutical products. The attitudes were not influenced or affected by the healthy lifestyle	Küster‐Boluda and Vidal‐Capilla (2017)
To study the behavior of customers toward functional foods and also to investigate the market for nutraceuticals in eastern Uttar Pradesh, India	A total of 200 consumers were selected from six different districts of eastern Uttar Pradesh, India	Consumer's interest in buying nutraceutical products was affected by factors like consumption pattern, health benefits, nutrition, cost, and availability of nutraceutical product	Jain et al. (2018)
To examine consumer's willingness to eat nutraceutical food	The survey was done through online mode in which a total of 810 respondents from the age group of 18–75 years were included out of which 49% were women consumers. Survey was done in Norway	Social pressure was the driving factor among consumers to eat nutraceutical products. Consumers were inspired to eat nutraceutical products and this also was an important factor	Nystrand and Olsen (2020)
To identify motivators and barriers of nutraceutical products	The survey was done among health‐conscious (HHC) and non‐health‐conscious (NHHC) consumers in Hungary	The survey suggested that the behavior of both the groups, i.e., HHC and NHHC toward nutraceutical products and their purchase is completely different. It was recommended that nutraceutical industries should undertake product promotional activities for both these groups in different manner	Papp‐Bata and Szakály (2020)
To identify crucial health issues which are commonly faced by the Hungarian population and acceptance of nutraceutical products in preventing these health concerns	A total of 1002 consumers were surveyed in Hungary	It was found that the senior citizens wanted to incorporate nutraceutical products into their diet. It was recommended that nutraceutical industries should take into account health issues like diabetes, obesity, and blood cholesterol while formulating nutraceutical products for senior citizens	Szakos et al. (2020)

### 3.1 Awareness of the benefits of nutraceuticals

The key factor, which attracts consumer attention and decides to buy behavior, is the health benefits offered by the nutraceutical product. In many published studies, authors have strongly stressed health benefits as an indispensable factor in attracting consumers toward nutraceutical products. Often, the perceived health well‐being made consumers buy nutraceutical products (Batty et al., 2013; Dickinson et al., 2014; Leung & Lum, 2011; Ralte et al., 2021; Sandmann et al., 2015; Seghiri & Essamri, 2019; Tangkiatkumjai et al., 2014; Teoh et al., 2021) but some of the consumers who were not sure of the beneficial effects of nutraceuticals ignored to buy them and took alternative medicines to overcome illness or diseases (Arthur et al., 2012; Jones et al., 2019; Tyler et al., 2008). The COVID pandemic has forced the consumer to learn about the health benefits of nutraceuticals and their contribution to boosting the immune system to fight infections (Ayseli et al., 2020; Kumar & Arora, 2023) through social media, google scientific published literature. This has also been reflected in the nutraceutical market growth and actual sale of nutraceutical products. For example, the sale of dietary supplements increased in the US by 44% in the first wave of COVID, whereas the demand for multivitamins increased by more than 50%. Similarly, there was high vitamin demand in the UK, and its sales increased by 63%. It can be said that this trend of increased nutraceutical demand was almost the same everywhere on the globe (Lordan, 2021). Consumers' attitudes toward buying nutraceutical products also changed a lot. The consumer, rather than believing in clinical trials, believed more in people who have tried the product and experienced positive effects. The changing approach also strengthened consumers' belief in nutraceutical products (Sinha & Efron, 2005). Fast forward to life in metros, where people do not get enough time to take diet and fulfill their hunger by eating fast food/processed food to have the perception that their diet is lacking nutrients required by their body and these people have now started taking nutraceuticals (Chen et al., 2011; Dickinson et al., 2014). On the other hand, people who think they are getting enough nutrients from their diet tend to ignore nutraceuticals (Downie et al., 2015). Older adults on medication, for one reason, are now trying to use nutraceuticals along with the medicines, as they believe that it may reduce the side effects caused due to long exposure to medicines (Hall et al., 2003; Kalichman et al., 2012). Consumers' attitude toward nutraceutical products closely relates to their choice, need, and circumstances, which will likely change with time. It is important to note that the impressive growth of the nutraceutical market in the current situation may not be a long term and the nutraceutical market may fall and return to the normal growth curve observed before the outburst of the COVID pandemic (Lordan, 2021).

### 3.2 Palatability

Palatability is closely related to the taste of edible products (Yeomans, 1998). The sensory attributes, most importantly, the nutraceutical product's taste, is crucial for consumers (Tahergorabi et al., 2015). Taste is a factor that varies with consumers and also with the age group. If the nutraceutical products fail to fulfill the taste of consumers, the product is ignored, and consumers start searching for the potential alternative (Arvanitoyannis & Krystallis, 2006; Tyler et al., 2008). A recent survey carried out in Kemerovo, Russia, by Kolbina et al. (2020) reported that along with the health benefits, the taste is also an important factor that decides the willingness of consumers to purchase nutraceutical products. The authors included 352 people in their survey, comprising people from the age group of 18–70 years, including both males and females. It was also reported that taste was the main factor for selecting nutraceutical products among younger consumers. In contrast, older consumers focused on taste as well as the price of the product. A study by Williams et al. (2004) also reported that the taste and smell of nutraceutical products were perceived as an additional advantage of nutraceutical products by consumers. The authors reported that the price of nutraceutical products was not crucial compared to the taste when deciding to buy a nutraceutical product. It is important to note that the palatability of nutraceutical products is vital in attracting young consumers toward them. However, due to the powerful health claims of some nutraceutical products, consumers who are health conscious may compromise on the palatability of nutraceutical products (Urala & Lähteenmäki, 2004).

### 3.3 Packaging

The packaging of nutraceutical products is the only point of attraction for consumers. Packaging is the first thing consumers notice before purchasing nutraceutical products, which applies to other food products. It is observed that more than 50% of decisions regarding purchasing products are made on the shelf (Abdalkrim & Hrezat, 2013). The design of packaging nutraceutical products should be such that it should attract the attention of consumers and should be unique among other products as such designs are mostly remembered and also purchased by consumers. Another important aspect related to packaging labels is giving essential information about the potential health benefits of nutraceutical products, which also affects consumers' decisions to buy nutraceutical products. Gutkowska and Czarnecki (2020) reported in the studies regarding consumer's attitudes toward packaging of functional foods that, along with the attractiveness of packaging, the nutritional information given on the packaging also played an important role in seeking consumers' attention and buying behavior of nutraceutical products. Oliveira et al. (2016) also reported in their studies that the label information given on the nutraceutical product is crucial in differentiating the nutraceutical products from other products, attracting consumers to buy the nutraceutical product. Another point that the nutraceutical industry should focus on is evaluating the environmental impact of packaging material. Packaging that is environmentally friendly and easily biodegradable will attract consumers and pass government regulations. Overall, the packaging of nutraceutical products is also a key factor in consumer perception and accordingly governs purchasing behavior of nutraceutical products.

### 3.4 Price and availability of the product

The consumer's ability to afford the nutraceutical product is also crucial in purchasing nutraceuticals. Generally, nutraceutical products are more expensive than conventional foods; hence, the higher cost directly influences consumers' willingness to buy. The higher cost of these products diverts consumers' attention, and consumers prefer to go with some alternatives at a lower price (Légaré et al., 2007; Patch et al., 2005; Sandmann et al., 2015). However, if the price of nutraceutical products is less than the conventional medicines available in markets, consumers will choose to buy nutraceutical products (Gardiner et al., 2007; Ibrahim et al., 2016). Of course, some consumers value health benefits more than the higher price of nutraceuticals, and such consumers willingly purchase nutraceutical products (Radman, 2005). Another obstacle to the demand and consumption of nutraceutical products is their availability in the market. The insufficient availability of nutraceutical products in the market has a negative impact on the consumer's perspective and purchasing attitude toward nutraceuticals. Suppose consumers find it difficult to access the nutraceutical product in the market. In that case, they go with the alternative conventional product, and that nutraceutical product loses its worth and purchasing capacity forever. Usually, consumers are more likely to go with products that are easily accessible and available in the market regularly. Suppose consumers lose interest in nutraceutical products merely because of their unavailability in the market or limited availability. In that case, it becomes difficult for nutraceutical industries to reattract consumers' attention toward that product by advertising or marketing, which additionally burdens nutraceutical industries. The nutraceutical product availability issue is seen prominently in developing nations compared to developed nations. Nutraceutical industries have typically failed to reach consumers in small cities, towns, and villages in developing and developed nations. As a result of which, these products are only available in metro cities. To attract consumers and expand the market, it is recommended that nutraceutical industries reach consumers even in interior parts of countries across the globe.

### 3.5 Social factors

The factors such as education, traditions, age, and gender also govern consumer purchasing behavior. Highly educated consumers are aware of the positive health benefits of nutraceutical products and show more interest in buying nutraceutical products than consumers who have less education or are uneducated (Ares et al., 2008). Kolbina et al. (2020) reported in their study that highly educated consumers, especially in the younger age group, had more knowledge about the health benefits of functional foods and showed positive attitudes toward buying nutraceutical products. Age and gender are also factors that differentiate buying behavior of consumers. Younger consumers who know the importance of nutraceuticals are more likely to search for related products on the shelf in markets. Besides, many studies also observed that female consumers are more likely to purchase nutraceutical products than male consumers. Çakiroǧlu and Uçar (2018) reported that the consumers willing to buy nutraceutical products were mostly females, the younger population, and educated individuals. It is also important to note that some studies showing the attraction of older consumers toward nutraceutical products are also seen. However, this may be confined to a limited number of older consumers, especially those suffering from diseases. For these persons, buying nutraceutical products is better than spending huge amounts of money in hospitals (Gupta & Prakash, 2015; Verbeke, 2005). Another contributing factor to the nutraceutical market is income. High‐income consumers tend to buy nutraceutical products that are usually costlier than conventional food. A recent study on the acceptance of pasta containing inulin showed that people with higher income preferred to go with such pasta containing health‐beneficial ingredients and were willing to pay for it compared to people with income below average level (Ivkov et al., 2018). Finally, traditional factors also greatly impact acceptance and willingness to purchase nutraceutical products (Siegrist et al., 2015). It is, thus, recommended to balance all the factors to arrive at the best marketing strategy to expand the nutraceutical market.

### 3.6 E‐commerce and advertising

E‐commerce has come into the limelight due to the COVID pandemic, where access to commercial establishments, including supermarkets, was hampered. Governments across the globe advised consumers to purchase through online shopping so that the spread of COVID infections can be lowered to a high extent (Hajizada et al., 2021; OECD Policy Responses to Coronavirus, 2020). The meteoric escalation in e‐commerce due to the COVID pandemic has been a boon to nutraceutical industries. Consumers who could not locate nutraceutical products on supermarket shelves could now order them online (Bell, 2021) using the available search engines. The shift to e‐commerce is a great opportunity for the nutraceutical industry to capture the market. Right from teenagers to old age, people are now interested in e‐commerce, which saves not only time but also money in terms of discounts consumers get online, and save on transport as well. Thus, nutraceutical industries should focus much on selling their product through e‐commerce and attracting consumers through discount offers, which will be a win−win situation for both consumers and industries. It is also important to note that the nutraceutical market is currently at its peak and continuously growing, but the postpandemic period may affect the nutraceutical industries. Thus, nutraceutical industries should adopt a healthy marketing/advertising strategy to keep growing to attract consumers. The first impression of the product decides the relationship between the consumer and the industry. The foremost look at the nutraceutical brand, its labeling, packaging, website, and information on the label will strongly impact the consumer's attitude toward the product (Goh et al., 2023). This first look usually decides whether that consumer will be with a specific nutraceutical product for their entire life.

### 3.7 Nutritional professionals

Many people routinely visit nutritional and healthcare professionals to seek advice for good health. The advice from these professionals regarding nutraceutical products plays an important role in deciding consumers' buying behavior. Many consumers will buy nutraceutical products if it is advised by nutritional professionals (Arcury et al., 2006). However, looking at the traditional practice, the nutritional professionals may advise consumers to go for conventional medicines rather than nutraceuticals. Hence, such consumers will be diverted from buying nutraceutical products. Légaré et al. (2007) reported that the nutritional professionals who were biased about the consumption of nutraceuticals prevented discussing the merits of nutraceuticals. This had an adverse effect on the consumers, which diverted consumers from buying nutraceutical products. Conversely, consumers are more likely to be motivated to buy nutraceutical products from gym instructors, healthcare staff, coaches, and dieticians. It is, thus, important that nutraceutical industries should focus on awareness programs, which they should conduct programs that will change the perception of nutritional and healthcare professionals toward nutraceutical products. Of course, some consumers do not come under the influence of professional suggestions, and their needs and requirements strongly govern their buying behavior. In such cases, the programs can also be aimed toward connecting with the prospective consumers directly.

Consumers across the globe have realized the importance of nutraceuticals which positively affect health, thus beneficially influencing the quality of life. It is generally observed that nutraceuticals are not considered a special food category by consumers. Instead, nutraceuticals are perceived as an additional alternative in the wide range of products available (Siro et al., 2008). Hence, consumers' willingness to choose between nutraceutical and conventional food products plays a crucial role in buying a product. In such cases, the packaging material and the information given on packaging material can greatly influence the consumers buying behavior if the same can provide important information about the nutraceutical product. The important information given on the food label, the package's size, the package's color, and the package's design help develop sensory expectations related to the product (Ares & Deliza, 2010; Becker et al., 2011). A study by Ares and Deliza (2010) showed that the packaging of nutraceutical products strongly influences the consumers' willingness to purchase the product. Consumers are attracted to nutraceutical products that are pocket‐friendly, budget‐friendly, palatable, easily consumed, and easily available in the local market. The factors governing consumers' willingness to buy nutraceutical product changes with the geographical region, local population, and their needs, gender of consumer, and the influence of e‐commerce. Hence, a single factor cannot decide the consumers' willingness to buy the product, and the consumer's buying behavior is a complex process involving many factors. These factors are relatively dependent on each other, and the study of these factors by entrepreneurs can help boost the nutraceutical market and product sales.

## 4 MARKET OF NUTRACEUTICALS

The nutraceutical market is rising continuously because of consumers' general awareness regarding nutraceuticals' health benefits. In addition, there is rapid growth in the urban population, and the increased risk of lifestyle‐related diseases is driving the increase in global growth. The market for nutraceuticals is classified based on factors such as type, form, and sales channel, which have been discussed.

### 4.1 Types of nutraceuticals

**FIGURE 2:**
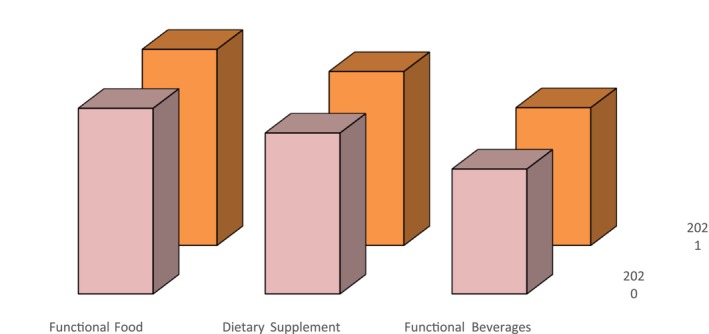
Recent Growth in nutraceutical market size.

Depending on the type of nutraceuticals, the market is segmented as functional beverages, functional food, and dietary supplements. Among these, functional foods occupied the highest share of the nutraceutical market in 2021 (Figure 2), with a profit‐sharing of about 40%, followed by dietary supplements and functional beverages. Functional foods are in demand due to their positive health benefits. Functional foods reduce the risk of diseases by enriching the body with vital nutrients and vitamins, strengthening the immune system (Munekata et al., 2021; Siro et al., 2008). The dairy and bakery segment of functional food has grown much faster and is estimated to grow in the coming year. As per the market analysis, the market value of the dairy segment of functional food was USD 48,831.8 million in 2019, which is supposed to cross the mark of USD 73,030 million by the end of 2027 (Functional Food Market, 2020). The main contributing factor is that functional dairy foods are easily available, and consumers know their health benefits. For example, in countries like India, probiotics are taken along with the diet to aid digestion (Markowiak & Ślizewska, 2017; Quigley, 2019). Probiotics grabbed the highest share of 28.3% among functional dairy foods in 2019. The increase in demand is across the globe, but the Asia pacific dominated the functional food market in 2019 and had an extortionate market share of 46.8% in 2019.

After functional foods, dietary supplements are the important type per the market share. The global market for dietary supplements was USD 140 billion in 2020, estimated to increase with a CGAR of 9%. Consumers are highly demanding multivitamins to manage their body weight and get energy. Vitamins were among the highest‐selling dietary supplements in 2020 in Western countries, with a share of about 31.4% in 2020. North America dominated the dietary supplement market with a revenue share of around 36% in 2020. There is a significant increase in the population in the Asia‐Pacific region, mainly in countries like India, China, Pakistan, Japan, and South Korea. In these countries, the dietary supplement is likely to accelerate and become one of the largest markets for dietary supplements by 2028 (Dietary Supplements Market Size, Share & Trends Analysis, 2022–2030).

Consumers' attention has also shifted toward healthy drinks/functional beverages in the last few years. Consumers prefer to drink functional beverages that satisfy their thirst and benefit consumers in terms of energy, soluble vitamins, minerals, and immune boosters. The functional beverage segment of nutraceuticals has also seen increased demand, as evident from the market figures. Energy drinks from functional beverages dominated the market with a contribution of around USD 44801 million in 2020, predicted to increase with a CAGR of about 5.1% and cross a figure of USD 75,365 million by the end of 2030 (Global News Wire, 2022). North America captured the major share of 39.5% of the functional beverages market in 2020. The contributing factor is that the region's higher aging population is more attracted to nutraceuticals due to their health issues.

### 4.2 Forms of nutraceuticals

The nutraceutical market is also divided according to the forms of nutraceutical products available. Nutraceutical products are available in the market as capsules, tablets, liquids, and powders. Consumers are attracted to the forms of nutraceutical products, which can be easily used and consumed. Capsules and tablets are among the most consumed forms of nutraceutical products. Capsules and tablets are smaller and occupy smaller space. Hence, it is easy to carry them and can be consumed anywhere and anytime. Liquids are packaged in packages that occupy much space and cannot be carried in wallets or purses. In contrast, powders also come in bigger packages and must be reconstituted at the time of consumption. Tablets and capsules dominated the nutraceutical market compared to powder and liquids globally. Nutraceutical industries should focus on introducing products in small and easily consumable packages, making it easier for consumers to carry and consume.

### 4.3 Nutraceutical market based on sales channel and region

The sales channels (SC) refer to a product's path to reach the consumer/final buyer. The probable outlets for consumers to get nutraceutical products are supermarkets, online shopping, pharmacy, and specialty stores. Pharmacies are places where medical drugs are sold which cure illnesses. Since nutraceuticals are also related to the well‐being of humans, nutraceuticals are more likely to be found in pharmacies. Indeed, many nutraceutical products were sold from pharmacies, with the pharmacy sector being the global leader in the nutraceutical market, followed by specialty stores. Due to the pandemic, e‐commerce has been flourishing and is expected to contribute to the global nutraceutical market in the future.

The demand for nutraceutical products substantially increased in the countries like China, India, Japan, and South Korea in recent years due to increased awareness among people and young consumers being attracted toward nutraceutical products like energy drinks, sports drinks, multivitamin supplements, protein powders, food enriched/fortified with bioactive/vitamins. The emergence of COVID also diverted consumers' attention toward nutraceutical products, especially to boost immunity and cope with the pandemic. The Asia‐Pacific region, hence, has dominated the nutraceutical market globally and is expected to keep the dominance in the future. The Asia‐Pacific market is estimated to reach USD 140,178.6 million by 2027 with a CAGR of 7.3%. Just after Asia Pacific comes the North American region, where the aging population is higher and prefers to take nutraceutical products to overcome age‐related problems. The sedentary work culture in this region has given rise to many health issues, including obesity. To overcome obesity, consumers are attracted to products like probiotics, prebiotics, essential fatty acids, proteins, and energy drinks, which are estimated to boost the nutraceutical market in North America in the coming years. North America's nutraceutical market is predicted to increase with a CAGR of 6.1% and reach USD 118.73 billion by 2027 (North America nutraceuticals market, 2022).

## 5 KEY PLAYERS IN THE NUTRACEUTICAL MARKET AND THEIR MARKET SHARE

Multinational food processing industries first introduced nutraceutical products in the market. Japan was the first country to introduce ‘Yakult’ as a probiotic beverage in 1935. Now, it is being sold in more than 40 countries around the globe. The food processing industries like Nestle, Unilever, and Danone started introducing nutraceutical products in the mid‐nineties. Nestle came up with LC1 yogurt in 1995, a probiotic yogurt intended to improve gut microbiota and aid digestion. Actimel Line followed the launch of Danone. Later after 5 years, in 2000, Unilever came up with ‘Becel‐margarine,’ made from plant lipids and containing no saturated fat/trans‐fat, and was claimed to reduce cholesterol. Companies like General Mills, Kellogg Co, Abbott Nutrition, Glaxo Smith, DuPont, Cargill, Amway, and Archer Daniel Midland Company are prominent nutraceutical market players. Many other companies also contribute to the nutraceutical market, but in small proportion and slowly growing. These companies should focus on diversifying their nutraceutical products and incorporating more natural ingredients to sustain in the market for a long. It is important to note that not all nutraceutical products will capture the market and continue to be attractive among consumers. It is interesting to share a failure story of AVIVA nutraceutical products of Novartis Consumer Health in Europe. The company launched nutraceutical products in Europe in 1999, but immediately, within 1 year, it had to withdraw its products due to lower sales (Menrad & Sparke, 2006). Usually, the product can capture consumers' attention if the health claims defined on the product are simple to understand and easily noticed. To grab the market successfully, nutraceutical industries should focus on products suitable for all age groups, as such products will be in demand by all the age groups. Naturally, sales of such products are expected to go up. One such product is ‘Yakult,’ which targets all age groups excluding infants, and is still the market leader in the category of probiotic beverages globally. In the specific context of the Indian market, nutraceutical industries are growing at a rate of 21% per year. Indian key players like Dabur, Baidyanath, Himalaya, Patanjali, Lifecare Neuro, and Dr. Reddy's have started focusing on formulating and delivering innovative nutraceutical products to cater to the demanding need of the Indian population, especially the younger generation. India is a big market for the nutraceutical industries to invest in and launch new products that use herbal ingredients/natural ingredients, protein powders, probiotic drinks, and fermented nutraceuticals. The health supplement sector of ‘Dabur India’ reported growth of around 31.9% in the financial year 2021. The revenue contribution to domestic Fast Moving Consumer Goods (FMCG) sales by the health supplement segment of Dabur India was 23.4% in 2021. Dabur occupies approximately 63% market share in the health supplement (Chyavanprash) sector (Dabur, 2021). ‘Amway’ is among the world's direct‐selling companies in over 80 countries. The Indian segment of Amway has registered a CAGR of more than 20% for the last 5 years, with the sale of nutraceutical products exceeding ₹100 crores. Amway has the highest share of 12% in the dietary vitamin supplement market, estimated to be around ₹9400 crores. The popular brand ‘Himalaya’ entered the nutraceutical sector some 10 years back with the launch of “HiOwna‐Jr” for kids, followed by the launch of HiOwna for adults and HiOwna Momz for pregnant and lactating mothers. With the launch of HiOwna Momz, Himalaya captured a 2.5% share of the nutraceutical market. An India‐based company ‘Patanjali Ayurved’ holds an appreciable market share in the Indian nutraceutical sector. The revenue of Patanjali Ayurved grew up in 2021 by 9% to ₹9872 crore. Abbott Nutrition dominates the infant formula market in the US, which occupies a 5% market share relative to its competitors in the US. The revenue and overall profit of almost all the players in the nutraceutical sector increased during the COVID period. After that, it experienced huge growth and demand for new and innovative products from consumers. A thorough study of the market, consumer needs, and continuous innovation in packaging, taste, and ingredients will help the big players in the nutraceutical market sustain and grow in the nutraceutical market in India and across the globe.

## 6 REGULATIONS GOVERNING NUTRACEUTICALS

A dietary supplement is the only term that is legally defined (Finley, 2016). US FDA defines ‘food’ as utilized for food or drink or components of any such article (Finley et al., 2014), and entry of the drug into the food market is restricted, which is an obstacle in the path of the nutraceutical sector not only in working on innovative products but also on claims made in the marketing of nutraceutical products. For example, Vitamin C is obtained majorly from citrus fruits by extraction, and foods are usually fortified with it to prevent deficiency‐related diseases like Scurvy. However, it is categorized under the head ‘drug’ defined by the FDA as a substance (other than food) used for treating or preventing diseases (FDA, 2014), and no drugs are allowed to enter the food market. Such ambiguous regulations are hurdles for nutraceutical industries to work on innovative products and expand their market in the USA and globally. In Europe, terms like ‘nutraceuticals,’ ‘dietary supplements, and other related terms are just a concept and are neither included in the food nor the drug category (Siro et al., 2008). There is no fixed regulatory skeleton for nutraceuticals in Europe (Bagchi, 2006). The European labeling legislation restricts assigning the properties like preventing, curing, and treating human diseases to food products. Health claims are dealt with at the national level; hence, there are no harmonized regulations on health claims in the European Union. European Union set up the European Commission Concerted Action on Functional Food Science in Europe (FUFOSE) to develop a scientific approach to support the formulation of nutraceutical products, i.e., functional foods having potential health benefits and reducing the risk of diseases in humans. FUFOSE made clear that functional food should not be in the form of pills or capsules but whole food, which is fortified with components beneficial for the well‐beings of humans (Diplock et al., 1999). In Asia Pacific, Japan was the first to cover Food for Specified Health Use (FOSHU) under Health Promotion Law (Yamada et al., 2008). The nutraceutical industries who need FOSHU approval are to give potential documents demonstrating the clinical evidence regarding claimed health benefits, and along with this, they are asked for the documents giving a clear picture of industrial production methods followed, types of equipment to be used, quality tests to be followed, packaging material used. This is not a compulsory approval but a voluntary approval system. Nutraceutical industries can market their nutraceutical product as ‘health foods' provided these products should not make any claim regarding the potential of these foods to reduce risk or treat certain diseases. In India, Food Safety and Standards Authority India (FSSAI) was passed in 2006 to regulate food products, including nutraceuticals, dietary supplements, and functional foods. According to this law, the products meant for special dietary use, like dietary supplements and functional food, cannot be taken as normal food; instead, these nutraceutical products can be made in the form of capsules, powders, tablets, liquid, or granules and should be administered orally. There should not be any claim made on the package of such product to treat/prevent the risk of diseases, and only limited health benefits are allowed to be shown as permitted by regulations governing the FSSAI act (Keservani et al., 2014). Thus, there are different regulations in different countries, which are an obstacle preventing the growth of the nutraceutical sector globally. There should be some harmonized regulations providing freedom in developing innovative products to cater to the need of consumers across the globe.

## 7 MINI SURVEY ON CONSUMER BEHAVIOR

### 7.1 Method followed

**FIGURE 3:**
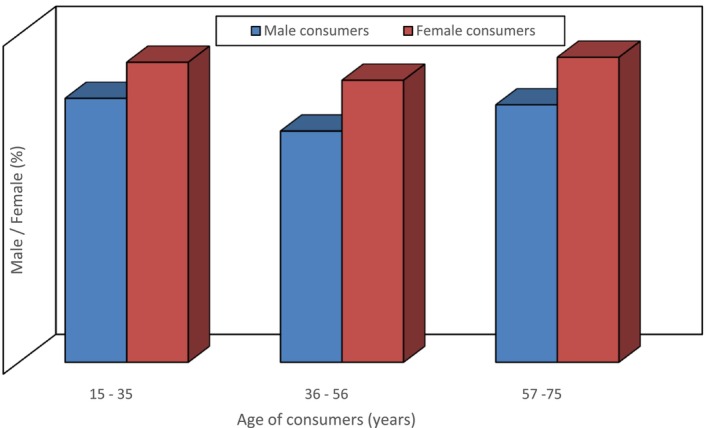
Distribution of male and female consumers according to their age.

A small survey was performed in Mumbai city of Maharashtra state in India. This short survey aimed to identify consumers' awareness regarding nutraceutical products available in the market. The survey was done in January 2022 among 400 consumers aged 15–75. Among these consumers, 40% were male, and 60% were female consumers (Figure 3). The ratio of female consumers was higher because, generally, it is observed that females are more approachable regarding nutrition‐related issues. The data have been collected exclusively by way of a personal questionnaire, and analysis of the data was done using SPSS Statistics software.

### 7.2 Conclusions are drawn from a survey

**FIGURE 4:**
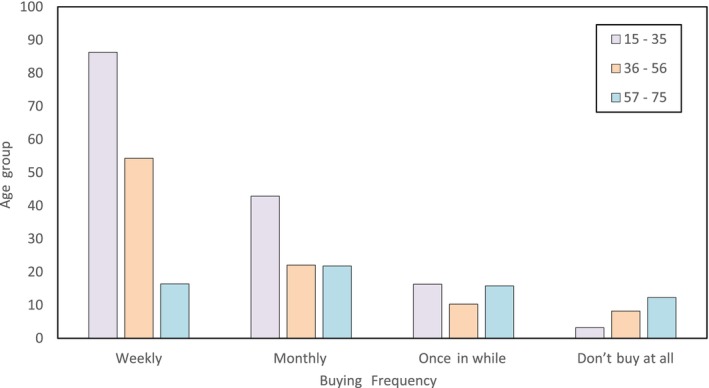
Nutraceutical products buying frequency among consumers of survey area.

**FIGURE 5:**
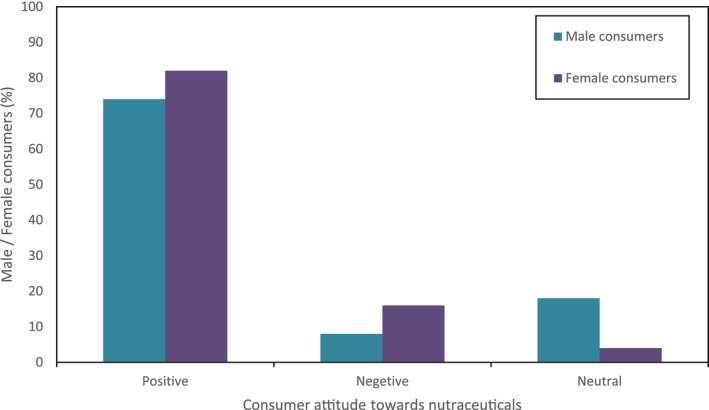
Consumers' attitude toward nutraceutical products.

In this survey, it was observed that consumers most commonly use nutraceutical products in the age group of 15–35 years, whereas these nutraceutical products were less popular in the age group of 55–75 years as the people were unaware of the health benefits of nutraceutical products. They feared certain side effects of these products on their bodies especially based on age and lack of awareness of the nutraceuticals. Among the types of nutraceuticals, functional food, i.e., probiotics, were mostly consumed by consumers daily, and the proportion was higher in females than males. Consumers believe that taking probiotics daily improves their metabolism and gut health. Sports, health, and energy drinks were also common among consumers aged 15–35. As noticed in the survey, consumers from the 15–35 age group preferred buying nutraceutical products once a week (Figure 4). The dairy products such as probiotics, prebiotics, whey proteins, Yakut, calcium‐rich dairy products, fortified dairy products, whey‐based beverages, and fermented dairy products were among the products which were common in almost all age groups and were in high demand by consumers. The awareness about nutraceutical products was less in older adults and mostly in females who were nonworking and housewives (Figure 5). These consumers were happy with their traditional foods and said that their traditional food, which they take in their diet, is a functional food for them.

Education also played an important role in deciding the response toward nutraceuticals. The highly educated consumers were aware of the health benefits of nutraceuticals and preferred to buy nutraceutical products every week. On the other hand, less educated people were unaware of nutraceutical products and preferred to go with their traditional foods. For example, female consumers aged 55 and above, who needed to be more highly educated and working, preferred to make probiotics at home rather than purchase from the market. According to them, the homemade probiotic dahi/yogurt contained more beneficial bacterial load than the probiotics available in the market.

The nutraceutical market is flourishing in India and is expected to grow in the coming future. However, nutraceutical industries should improve their marketing strategies to attract consumers from all age groups. Advertisements must be designed to tell consumers about health benefits so that even an uneducated person should be attracted to their product. Currently, as seen from the survey results, young consumers are dominating the nutraceutical market in Mumbai and are ready to welcome new nutraceutical products launched by the nutraceutical industries. A good and proper marketing strategy of advertisement coupled with continuous innovation in existing products and the launch of new nutraceutical products will be a boon to the nutraceutical market in India.

Similar surveys have been reported in the literature to confirm the importance of the obtained information based on the survey. A recent study by Kolbina et al. (2020) identified the distribution of the target consumers' toward functional food products in Kemerovo city, Russia. The authors surveyed 352 people aged 18–70 years, with 55% women and 45% men. The authors concluded that consumers aged 18–40 years of knowing products showed a willingness to purchase functional products irrespective of their high prices. Consumers' key criteria for purchasing functional food were its taste and therapeutic and prophylactic properties. Consumers aged 50–70 years paid more attention to the quality and the price of functional products. In summary, the authors concluded that the consumers in the age group of 18–40 years having higher education were among the potential buyer of functional foods in Kemerovo city, Russia, similar to the current findings in Mumbai, India.

## 8 CONCLUSIONS

Desk‐bound lifestyle in the 2lst century has given rise to many noncommunicable diseases (NCD) such as diabetes, obesity, and heart‐related diseases across the globe. Due to the pandemic, work‐from‐home/online work has been an add‐on to health‐related issues. Nutraceutical products have emerged as a ray of hope to prevent these NCD and mental health issues and help to improve human health. However, many factors govern consumers' behavior toward buying nutraceutical products. Crucial factors such as health benefits and safety concerns related to these products are preventing consumers from buying these products. However, the COVID pandemic has been a boon to the nutraceutical market, as evident from the figures showing growth of the nutraceutical sector, especially functional foods which have grown from USD 161.99 billion in 2020 to USD 171.25 billion in 2021. The short survey in Mumbai city of India revealed that the health benefits and the knowledge about nutraceutical products were among the pragmatic determinants of acceptance of nutraceutical products. Young consumers and female consumers were found to be highly attracted to these nutraceutical products. In contrast, old people with health problems preferred buying nutraceutical products frequently though some old people preferred taking their traditional food along with the prescribed medicines. The main reason for not preferring nutraceuticals was the knowledge gap and sociodemographic factors. In summary, nutraceutical industries should adopt simple and informative advertisement patterns and work on innovative products that could be easy to carry and eat. They should be easily available not only in metro cities but in small towns as well. The suggested approach will likely keep the nutraceutical market blooming even after the pandemic.

## AUTHOR CONTRIBUTIONS

**Harsh Jadhav:** Conceptualization (equal); data curation (equal); formal analysis (equal); resources (equal); software (equal). **Shyam S. Sablani:** Formal analysis (equal); methodology (equal); software (equal); writing – original draft (equal). **Parag Gogate:** Data curation (equal); investigation (equal); software (equal); visualization (equal). **Uday Annapure:** Methodology (equal); supervision (equal); visualization (equal); writing – original draft (equal). **Federico Casanova:** Software (equal); validation (equal); writing – review and editing (equal). **Gulzar Ahmad Nayik:** Formal analysis (equal); methodology (equal); supervision (equal); writing – review and editing (equal). **Kamal Alaskar:** Investigation (equal); software (equal); validation (equal); visualization (equal). **Nazmul Sarwar:** Funding acquisition (equal); investigation (equal); software (equal). **Irfan Ahmad Raina:** Data curation (equal); formal analysis (equal); software (equal); supervision (equal). **Seema Ramniwas:** Data curation (equal); methodology (equal); software (equal); writing – review and editing (equal). **Amin Mousavi Khaneghah:** Software (equal); supervision (equal); validation (equal); writing – review and editing (equal).

## FUNDING INFORMATION

None.

## CONFLICT OF INTEREST STATEMENT

The authors confirm that they have no conflicts of interest concerning the work described in this manuscript.

## Data Availability

Data are available from the corresponding author and can be obtained upon request.
